# Electrochemical Characterization and Determination of Phenol and Chlorophenols by Voltammetry at Single Wall Carbon Nanotube/Poly(3,4-ethylenedioxythiophene) Modified Screen Printed Carbon Electrode

**DOI:** 10.1155/2015/459246

**Published:** 2015-11-01

**Authors:** Negussie Negash, Hailemichael Alemu, Merid Tessema

**Affiliations:** ^1^Department of Chemistry, Addis Ababa University, P.O. Box 1176, Addis Ababa, Ethiopia; ^2^Department of Chemistry and Chemical Technology, National University of Lesotho, P.O. Roma 180, Roma, Lesotho

## Abstract

Screen printed carbon electrode (SPCE) has been modified with single wall carbon nanotube/poly(3,4-ethylenedioxythiophene) (SWCNT/PEDOT) composites for the determination of phenol and chlorophenols (phenol, 4-chlorophenol, 2,4-dichlorophenol, and 2,4,6-trichlorophenol). The effect of the modifiers on the electrode characteristics was evaluated and the responses were optimized for the voltammetric determination of phenol and chlorophenols. The parameters affecting the responses such as pH, scan rate, and stability were studied. The analytical performance of the SWCNT/PEDOT/SPCE using cyclic voltammetry was tested and found to be impressive. Under these conditions, the designed electrode showed a good performance for the voltammetric measurements of the phenolic compounds. The modified SPCE, when it is compared with other enzymatic and nonenzymatic sensors, showed a wider dynamic range for the detection of the phenolic compounds. The modified SPCE was used for the quantification of phenol in water samples. The results suggest that the method is quite useful for analyzing and monitoring phenols and chlorophenols.

## 1. Introduction

Screen printing technique seems to be one of the most promising approaches allowing simple, versatile, rapid, and low cost sensor/biosensor production [1]. In addition, the screen printed electrode (SPE) has enabled the production of modern sensors, which can be incorporated in portable systems, an important requirement of detection methods for the direct on-field analysis of a sample without changing of the natural environmental conditions [2]. A disposable screen printed sensor can be prepared from noble metals, such as Au and Pt, but these require high firing temperatures [3]. Inks based on carbon have very low firing temperature and can be printed on plastic substrates. Therefore, the most commonly used material for SPE fabrication is carbon ink and thus named as screen printed carbon electrode (SPCE).

The applications of sensors based on screen printed electrodes for the detection of heavy metals in biomolecules, pesticides, antigens, and ions have been well documented [4–7]. Electrochemical sensors based on screen printed electrodes are simple to be used with in situ screening devices, since all the equipment needed for the electrochemical analysis is portable. They have all the basic performance characteristics of sensors, among them the requirement of minimum sample preparation, simplicity of the apparatus, fast responses, low cost, and miniaturization with new technologies [7, 8].

Disposable electrochemical sensors based on SPCE for the determination of trace levels of pollutants and toxic compounds in environmental and biological samples are also attractive because of economic considerations and the design of SPCE can be changed with respect to the requirements for a specific analyte. In addition, the surface of SPCE can be easily modified to fit multiple purposes with regard to different pollutants and to achieve a variety of improvements [9, 10].

Phenolic compounds usually coexist in environmental samples and are toxic to animals and aquatic organisms. Therefore, the rapid in situ determination of phenolic compounds and their derivatives is an important environmental challenge. SPCE based electrochemical sensors and biosensors have led to a low cost, simple and sensitive analytical method for the identification and determination of phenolic compounds. In fact, the electrooxidation products of phenol would passivate the surface of SPCEs and hinder the electron transfer process. To alleviate such problems, different configurations for the design of screen printed enzymatic biosensors involving different techniques for enzyme immobilization have been attempted [6, 11]. Enzymes have been widely used in the preparation of biosensors for the analysis of phenolic compounds and have allowed the measurement to be performed at a low applied potential with significant reduction of interference. Reports of studies performed at chemically modified SPCEs for the determination of phenolic compounds include nanomaterial modified SPCE [12], a nanocomposite-modified SPCE coated with multiwalled carbon nanotubes (MWCNT), electrodeposited gold nanoparticles (AuNPs) [2], and an electrochemically pretreated SPCE [13]. Enzyme based SPCE still suffers from the rapid loss of the biochemical activity of the enzyme and leakage into the solution. An electrochemically pretreated SPCE offers simple analysis of phenolic compounds but is limited to diols which suffers less in fouling due to the electrochemical passivation of phenol oxidation products [6].

On the other hand, conducting polymers such as polyaniline, polypyrrole, and polythiophene are suitable materials as electrocatalysts for detecting organic and biological molecules [14]. Due to the continued interests in exploring the applications of polymeric layers in electroanalysis, poly(3,4-ethylenedioxythiophene), PEDOT, the conducting polymer, has been used to modify SPCE. For example, the electrochemical investigation of neutral acetaminophen (ACAP) at PEDOT modified SPCE shows favorable electrocatalysis for ACAP in aqueous media [15]. Tyrosinase based biosensors with PEDOT/Poly(styrene sulphonate) modified SPCE allowed the detection of phenolic compounds with significantly improved sensitivity and wider linear range when compared to Tyrosinase immobilized on screen printed carbon electrode [16].

The incorporation of nanomaterials in the screen printed electrodes has attracted attention in the development of electrochemical sensors. Carbon nanotubes that are molecular-scale tubes of graphitic carbon present a singular structure and dimensions together with unique electronic, chemical, and mechanical properties. [17–19]. As electrode materials, CNT facilitate electron transfer between the electroactive species and the electrode. Consequently, the sensors have fast response and signal enhancements. One of the carbon nanotubes is SWCNTs, which are one-dimensional conductor with all electrons moving in an atomic layer having surface atoms. Recently, a novel screen printed single wall carbon nanotube electrode cast on flexible polyester substrates was fabricated and electrochemically characterized. The electroanalytical performance of this sensor was applied towards the detection of potassium ferrocyanide(II), dopamine, hydrazine, and capsaicin [20].

At present, there is a great interest in exploiting the exciting properties of these CNTs by incorporating them into some form of polymer matrix. Indeed, a large number of preparation techniques have been attempted to form CNTs/polymer composites with enhanced mechanical and electrical properties [21–25]. Although a number of electrode materials based on CNT-conducting polymer composite were used for the voltammetric determination of phenol and its derivatives, very few researchers investigated the use of CNT-conducting polymer composites modified SPCE electrodes [26].

The preparation of new type of composite materials that have distinct properties as modifiers of SPCE for the electrocatalytic oxidation of phenol and chlorophenols was the aim of this study. To the best of our knowledge, the modification and application of SPCE with SWCNT/PEDOT as voltammetric sensor for phenol and chlorophenols had not been reported so far. The study thus focused on the development of screen printed carbon electrode modified with the composites of SWCNT/PEDOT for the investigation of the electrochemical behavior and determination of phenol and chlorophenols.

## 2. Experimental

### 2.1. Chemicals and Reagents

SWCNT (mixtures of metallic and semiconducting), 3,4-ethylenedioxythiophene (EDOT) monomer, and tetrabutylammonium perchlorate were purchased from Sigma-Aldrich (Prestige Laboratory Supplies Company, Durban, South Africa). Sodium acetate, phenol, 4-chlorophenol, 2,4-dichlorophenol, and 2,4,6-trichlorophenol were purchased from Fluka (Shalom Laboratory Supplies Company, Durban, South Africa) which were used in the experiments. Other chemicals were from Chemoquip (Johannesburg, South Africa). All chemicals were of analytical grade and were used without further purification. All solutions were prepared using ultrapure water of resistivity 18.2 MΩ-cm obtained from ELGA PURELAB Option-Q (UK) water purification system. SWCNT was dispersed in N,N-dimethylformamide (1 mg SWCNT : 1 mL DMF) and sonicated for 1 hour to achieve well-dispersed suspension. EDOT monomer of concentration 0.01 M was dissolved in acetonitrile that contained 0.1 M tetrabutylammonium perchlorate supporting electrolyte. Fresh solutions of 10^−2 ^M phenol in water and 10^−3 ^M of 4-chlorophenol, 2,4-dichlorophenol, and 2,4,6-trichlorophenol were prepared separately in 50% water/methanol mixture and diluted to the required concentrations. Sodium acetate of concentration 0.1 M was used as a buffer solution and the required pH was adjusted by adding either acetic acid or sodium hydroxide solution.

### 2.2. Preparation of Modified Electrodes

SWCNT modified SPCE was prepared by depositing 10 *μ*M dispersion of SWCNT onto the surface of the bare electrode followed by drying it at room temperature for an overnight and is denoted as SPCE/SWCNT. PEDOT modified SPCE was prepared by electropolymerization of 0.01 M EDOT on the surface of SPCE. The polymer film was washed with water to remove the supporting electrolyte and the unreacted monomer. Electrochemical polymerization of EDOT on SPCE was performed by 10 cycles of cyclic voltammetry scanning the potential from −0.9 to +1.5 V at a scan rate of 10 V s^−1^ [16]. Different PEDOT modified electrodes were prepared by varying the number of potential cycles (5, 10, 20, 40, and 60) to obtain films of varying thicknesses. The PEDOT modified film electrode is denoted as SPCE/PEDOT.

Further, two types of modified electrodes, namely, SPCE/SWCNT/PEDOT and SPCE/PEDOT/SWCNT, were prepared. SPCE/SWCNT/PEDOT was prepared by coating first the SWCNT suspension on the SPCE followed by polymerization of EDOT and SPCE/PEDOT/SWCNT was prepared by polymerizing first EDOT on the SPCE and then casting the SWCNT suspension. Prior to the experimental measurements, the modified electrodes were first activated by 20 repetitive potential cycling between 0 and 1.2 V in 0.1 M acetate buffer solution as the base electrolyte to obtain steady state CV responses at a scan rate of 50 mV s^−1^.

### 2.3. Instrumentation

Electrochemical measurements were carried out in a single-compartment cell using CHI 840C Work Station (USA) controlled by Dell, personal computer. A conventional three-electrode system was used in the voltammetric measurements. Disposable SPCE (DRP110) consisting of carbon ink based working electrode (4 mm diameter), a carbon counter electrode, and an Ag reference electrode was purchased from DropSens Inc. (Spain) for the electrochemical measurements. The working electrode was modified to obtain SPCE/SWCNT, SPCE/PEDOT, SPCE/SWCNT/PEDOT, and SPCE/PEDOT/SWCNT. A sensor connector cable CAC (DropSens Inc., Spain) allowed for connecting the electrodes to the CHI Work Station.

**Figure 1:**
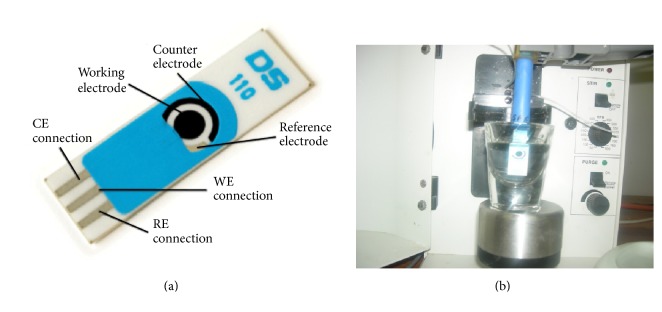
The electrochemical cell: (a) screen printed carbon electrode, counter carbon electrode, and Ag reference electrode; (b) connector cable with the electrochemical cell.

The electrochemical cell volume in all experiments was 10 mL, and all measurements were performed at room temperature. The pH of the solutions was measured using Jenway 3345 pH/Ion meter and for sonication Branson 2510 (USA) ultrasonic water bath was used.  Figure 1 shows the screen printed carbon electrode, counter carbon electrode, Ag reference electrode, and the connector cable with the electrochemical cell.

## 3. Results and Discussion

### 3.1. Electrochemical Behavior of Phenol at Screen Printed Carbon Electrode and Modified Screen Printed Carbon Electrodes

Screen printed carbon electrodes (SPCEs) were modified with SWCNTs, PEDOT, and PEDOT/SWCNTs composites. SWCNTs were added to the modified electrode for the detection of phenol in order to improve its sensitivity, stability, and mechanical strength. CV was chosen in this work because it provides a better way to control the growth of the polymer film and to avoid overoxidation. Different PEDOT modified electrodes were prepared by varying the number of potential cycles: 5, 10, 20, 40, and 60 to obtain films of varying thicknesses between the potential window −0.9 and +1.5 V. The extent of electropolymerization increased with increasing the cycle. A plot of current versus number of cycles showed that the polymer film prepared with 10 cycles had the best current response (figure not shown).

**Figure 2:**
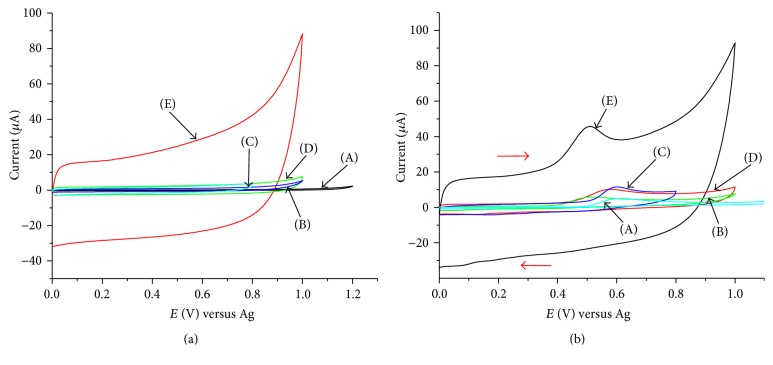
Cyclic voltammograms for the base electrolyte (a) and 1 × 10^−4 ^M phenol (b) at SPCE (A), SPCE/SWCNTs (B), SPCE/PEDOT (C), SPCE/PEDOT/SWNTs (D), and SPCE/SWCNTs/PEDOT (E); scan rate: 0.050 V s^−1^; 0.1 M acetate buffer pH 6.

The voltammetric responses of bare screen printed and modified screen printed electrode for 10^−4 ^M phenol, in 0.1 M acetate buffer, pH 6, were studied using CV.  Figure 2 compares cyclic voltammograms of the buffer solution and 10^−4 ^M phenol at SPCE, SPCE/SWCNTs, SPCE/PEDOT, SPCE/PEDOT/SWCNTs, and SPCE/SWCNTs/PEDOT.

In the presence of only the base electrolyte, Figure 2(a), the voltammogram of SPCE/SWCNTs/PEDOT (E) shows large background current which is an indication of the effective modification of the electrode which is exhibited by large double-layer charging current. Compared with the bare SPCE (A), the SWCNTs modified SPCE (B) and the PEDOT modified SPCE do not show significant difference in their background currents.

Figure 2(b) shows the oxidation peaks of 1 × 10^−4 ^M phenol at the different five electrodes. The peak potentials and peak currents of phenol at the five electrodes are presented in Table 1. As shown in the table, the peaks of phenol appear at different potentials and the lowest peak potential is 509 mV for the electrode SPCE/SWCNTs/PEDOT which is 48 mV lower than the unmodified SPCE. Comparison of the peak currents of phenol at these electrodes also shows that the maximum peak current is 21.38 *μ*A corresponding to the electrode SPCE/SWCNTs/PEDOT. This value is five times larger than the value recorded for the unmodified SPCE. The SPCE/PEDOT and SPCE/PEDOT/SWCNTs also show enhancement in the peak current of phenol as twice as that of SPCE, whereas the current for SPCE/SWCNTs is not significantly different from SPCE.

The modification of the screen printed carbon electrode with SWCNTs followed by EDOT polymerization (SPCE/SWCNTs/PEDOT) shows much stronger electrocatalytic activity for the oxidation of phenol resulting in an increase of the peak current and negative shift of the peak potential. Thus, it can be concluded that the increase in peak currents and shift in peak potential are the result of the synergic effect for the incorporation of SWCNTs into the PEDOT polymer [27].

**Figure 3:**
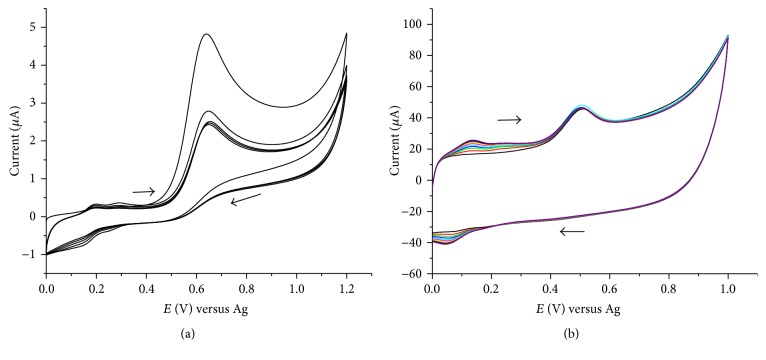
Repetitive cyclic voltammograms for 1 × 10^−4 ^M phenol in a 0.1 M acetate buffer solution, pH 6 at bare SPCE (a) and SPCE/SWCNTs/PEDOT (b); scan rate of 0.05 V s^−1^.

The electrocatalytic activity of the SWCNT, PEDOT, and composite film modified SPCE is demonstrated by running repetitive cyclic voltammograms for 1.0 × 10^−4 ^M phenol in pH 6.0 acetate buffer solution recorded at five different working electrodes.  Figure 3(a) shows the repetitive ten cyclic voltammograms at the bare SPCE. The oxidation of phenol appears at 639 mV and after the first cycle the current decreases substantially and becomes stable after the second cycle. A continuous decrease in the peak current was observed with a gradual positive shift in the peak potential at the SPCE/SWCNTs for ten successive cycles signifying a fouling of the electrode surface as a result of the formation of nonconducting oxidation product of phenol. Similar gradual positive potential shifts without much decrease in current were observed at SPCE/PEDOT and SPCE/PEDOT/SWCNTs (figures not shown).  Figure 3(b) shows the oxidation of phenol at the SPCE/SWCNTs/PEDOT for ten repetitive cyclic voltammograms. The peak potential and the peak current remain the same for all cycles indicating the absence of fouling of the electrode or change in the morphology of the surface of the electrode. The low oxidation peak potential and the enhanced peak current observed at this electrode clearly demonstrates that SPCE/SWCNTs/PEDOT composite film efficiently promotes the kinetics of the electrochemical reaction, which is probably caused by the synergistic effect of the electrocatalytic property of SWCNTs and PEDOT [27] and the contribution from the unique electronic and chemical properties of CNTs [17, 18]. CNT modified SPCEs are mechanically strong and provide excellent attachment to the ink surfaces [2, 27]. These results indicate that the SPCE/SWCNTs/PEDOT composite film modified electrode can not only detect phenol, but also enhance the detection sensitivity. Furthermore, the modified electrode was used and applied for the analysis of chlorinated phenols.

### 3.2. Effect of pH

**Figure 4:**
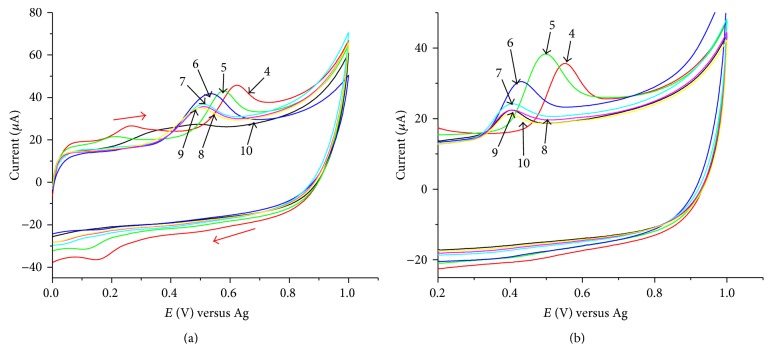
Cyclic voltammograms for 1.0 × 10^−4 ^M phenol (a) and 2,4,6-trichlorophenol (b) at SPCE/SWCNTs/PEDOT in 0.1 M acetate buffer solution of varying pH; scan rate: 0.05 V s^−1^.

As the solution pH is an important factor affecting the electrochemical reactions of phenolic compounds in aqueous solution, cyclic voltammetry was performed to investigate the effect of solution pH on the electrochemical response of 1 × 10^−4 ^M phenol and chlorophenols at the SPCE/SWCNTs/PEDOT. The effect of pH on the anodic peak current of phenol and chlorophenols were examined in the pH range 4 to 10 using 0.1 M acetate buffer and the representative figures are depicted in Figure 4. The oxidation peak current for phenol increased up to pH 6 and gradually decreased as the pH increased, Figure 4(a). The same trend was also observed for 4-chlorophenol (figure not shown). For 2,4,6-trichlorophenol, the peak current increased up to pH 5 and then gradually decreased from pH 6 to 10, Figure 4(b). Similar current versus pH profile was also observed for 2, 4-dichlorophenol (figure not shown). Thus, pH 6.0 was chosen as the optimum pH value for the electrochemical determination of phenol and 4-chlorophenol while pH 5 was chosen for 2,4-dichlorophenol and 2,4,6-trichlorophenol.

**Figure 5:**
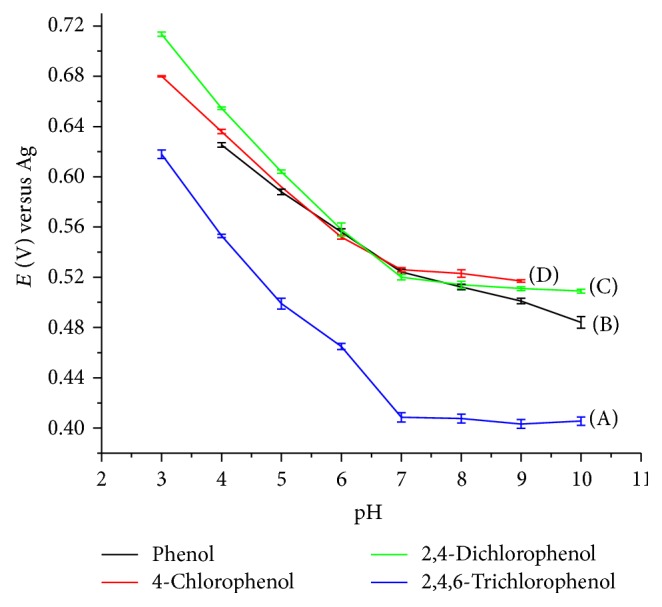
The effect of pH on peak potential of 1 × 10^−4 ^M: 2,4,6-trichlorophenol (A), phenol (B), 2,4-dichlorophenol (C), and chlorophenol (D); other conditions are as in Figure 4.

The variation of the peak potential as a function of pH for the oxidation of phenol and chlorophenols are depicted in Figure 5. It can be observed that the peak potentials shifted negatively with the increase in the pH of the supporting electrolyte solution, Figure 5. Within the range of pH 3 to 7, the anodic peak potentials of phenol and chlorophenols varied linearly with pH of the solution and with shifts to more negative values.

The linear regression equation for phenol oxidation was *E*_*p*_(*V*) = −0.0599pH + 1.228 (*R* = 0.9784). The 59 mV pH^−1^ slope indicated that the electrochemical oxidation of phenol was two-electron two-proton process. In view of the above findings, it is obvious that the oxidation of phenol and chlorophenols is a favorable process in acidic medium.

### 3.3. Effect of Scan Rate

**Figure 6:**
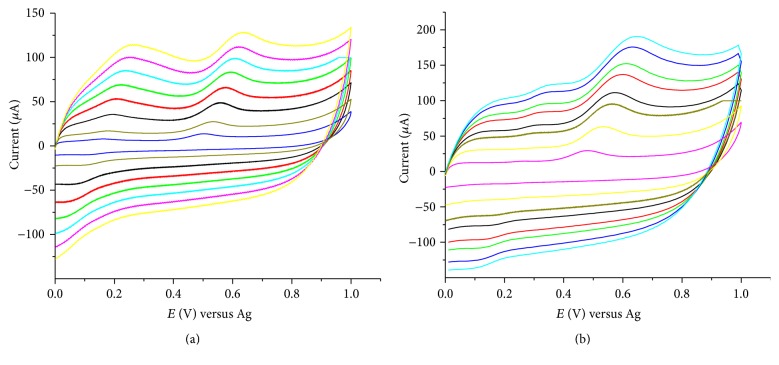
Cyclic voltammograms for 1 × 10^−4 ^M phenol (a) and 2,4-dichlorophenol (b). For phenol, scan rate: 0.02, 0.05, 0.10, 0.15, 0.20, 0.25, and 0.30 V s^−1^, pH 6; for 2,4-dichlorophenol, scan rat: 0.02, 0.05, 0.08, 0.1, 0.13, 0.15, 0.18, and 0.20 V s^−1^, pH 5.

**Figure 7:**
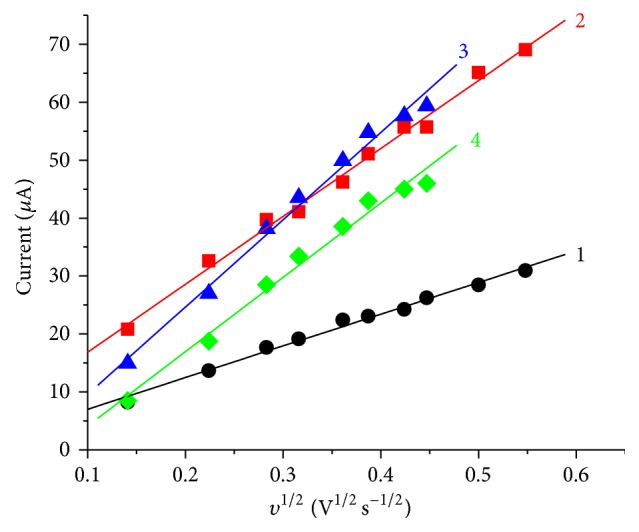
Plot of oxidation peak current as a function of the square root of the scan rate: phenol (1), chlorophenol (2), 2,4-dichlorophenol (3), and 2,4,6-trichlorophenol (4) each with a concentration of 1 × 10^−4 ^M.

The effect of scan rate on the cyclic voltammetric responses at SPCE/SWCNTs/PEDOT was investigated using 0.1 M acetate buffer solutions.  Figure 6 depicts the representative cyclic voltammograms obtained for 1 × 10^−4 ^M phenol and 2,4-dichlorophenol studied at SPCE/SWCNTs/PEDOT in the scan rate range of 0.02 to 0.40 V s^−1^. The oxidation peak of phenol is observed in the potential range of 0.5–0.6 V shifting more towards positive potential with increasing scan rate. Another anodic peak is also observed at about 0.2 V that that has a corresponding reduction peak at about 0.1 V. The electrooxidation pathway of phenol at a modified electrode is different from that of unmodified electrode [24, 28]. At modified electrode, the oxidation peak of phenol is followed by the formation of paraquinone/hydroquinone redox couple and or orthoquinone/catechol redox couple. The redox peaks that are observed in the potential region of 0.1–0.2 V presumably could be due to the formation of ortho-quinone/catechol.  Table 2 summarizes the oxidation peak currents of phenol and chlorophenols at different scan rates. A plot of the oxidation peak currents as a function of the square root of the scan rate is shown in Figure 7. From the plots of the peak current versus the square root of the scan rate (*υ* ^1/2^), it was found that the peak current is proportional to the square root of the scan rate in the range of 0.02 to 0.35 V s^−1^ for phenol and chlorophenol and in the range of 0.02 to 0.25 V s^−1^ for 2,4-dichlorophenol and 2,4,6-trichlorophenol with an average of 0.998 regression coefficient. At relatively higher scan rate for 2,4-dichlorophenol and 2,4,6-trichlorophenol distortion of the CV, waves were observed. This effect often sets the limits of maximum useful scan rate and the charging current becomes relatively more important at faster scan rates [29]. The linear dependence of the oxidation peak currents on the square root of the scan rate indicates that the electrochemical reactions are diffusion controlled processes, similar to previous studies of the electrochemical oxidation of phenol and chlorophenols [13, 15, 30].

### 3.4. Electroanalytical Applications of the SPCE/SWCNTs/PEDOT Sensor

**Figure 8:**
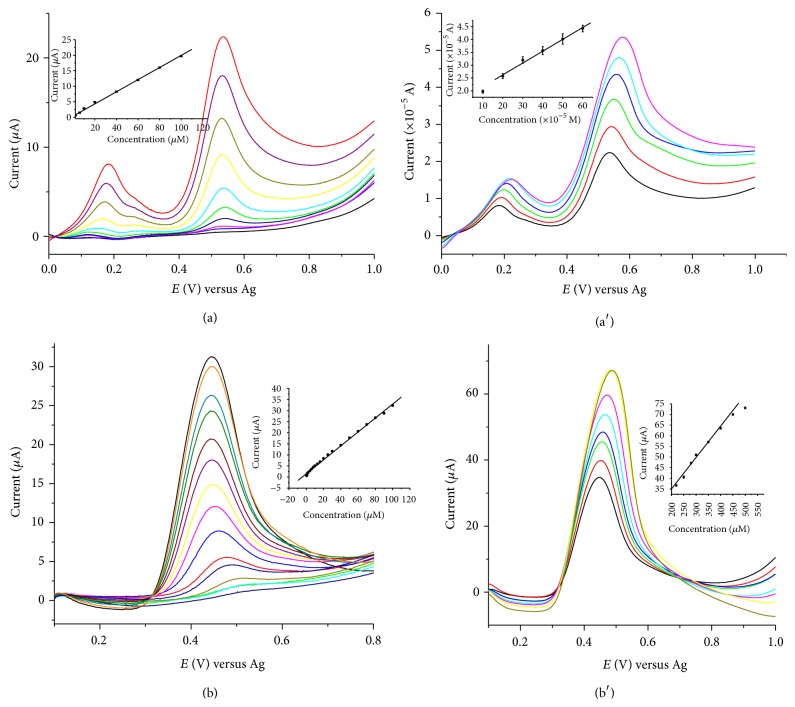
Linear scan anodic voltammograms at SPCE/SWCNTs/PEDOT: for phenol (a) and (a′) (at pH 6, concentration range: 0.6–100 *μ*M and 200–600 *μ*M, resp.) and for 2,4,6-trichlorophenol (b) and (b′) (at pH 5, concentration range: 0.4–100 *μ*M and 220–500 *μ*M, resp.); scan rate 0.05 V s^−1^. The insets are the corresponding calibration plots.

The current responses of SPCE/SWCNTs/PEDOT for the concentration of phenol, 4-chlorophenol, 2,4-dichlorophenol, and 2,4,6-trichlorophenol were studied using linear scan anodic voltammetry measurements at the optimized conditions. Increase in the current responses was observed as the concentrations of phenol and chlorophenols were increased. All phenols and chlorophenols gave two linear ranges when the oxidation current responses were plotted as a function of their concentrations.  Figure 8, for example, depicts the linear scan anodic voltammograms for phenol (a) and (a′) for the concentration ranges 0.6–100 *μ*M and 200–600 *μ*M, respectively, and for 2,4,6-trichlorophenol (b) and (b′) for the concentration ranges 0.4–100 *μ*M and 220–500 *μ*M, respectively. The calibration curves obtained from the voltammograms (inset curves), Figure 8, show the linear relationships between the current (*μ*A) as a function of the concentrations (*μ*M) of phenol and 2,4,6-trichlorophenol. Analytical parameters obtained from the response of the linear range of the calibration curves at the SPCE/SWCNTs/PEDOT electrochemical sensor are summarized in Table 3.

The analytical performance of the SPCE/SWCNTs/PEDOT evaluated from the voltammetric responses has been compared with other screen printed electrochemical sensors reported recently, Table 4. Characteristics such as type of electrode, linear range, sensitivity, and limit of detection achieved were compared. The PEDOT/SWCNTs composite based screen printed carbon electrode exhibits a higher electrochemical activity compared with other screen printed electrodes. In addition to the simplicity of the preparation of the sensor wider or similar dynamic ranges, low detection limits and comparable sensitivity were obtained compared to the recently reported sensors/biosensors.

### 3.5. Recovery Study

The modified sensors prepared were found to be useful in the voltammetric determination of phenol in distilled and tap water. Recovery studies were carried out using three water samples each with a concentration of 0.1 M acetate and 10 *μ*M phenol solution and three tap water samples each with a concentration of 0.1 M acetate and 10 *μ*M phenol solution. Different concentrations of phenol were then spiked to each sample solution and the corresponding current responses were recorded. From the calibration curve for phenol (0.6–600 *μ*M), the total concentration of phenol in each sample was found as shown in Table 5. To evaluate the validity of the results, recovery values were calculated from the percentage ratio of found phenol concentration to the total phenol concentration and the values are found in the range of 98.85% to 104.46%, which is in the acceptable range. The results obtained confirm that the designed modified electrode can successfully be used for the quantitative determinations of phenols and chlorophenols using voltammetry.

### 3.6. Reproducibility Study

The reproducibility of the results was examined by successive ten CV runs of 1 × 10^−4 ^M phenol and chlorophenols using the optimum conditions given previously. The relative standard deviation (RSD) was calculated and it was found to be 0.65% for phenol, 0.817% for 4-chlorophenol, 2% for 2,4-dichlorophenol, and 1.12%. The obtained results indicate the good stability of modified electrode.

### 3.7. Interference Study

**Figure 9:**
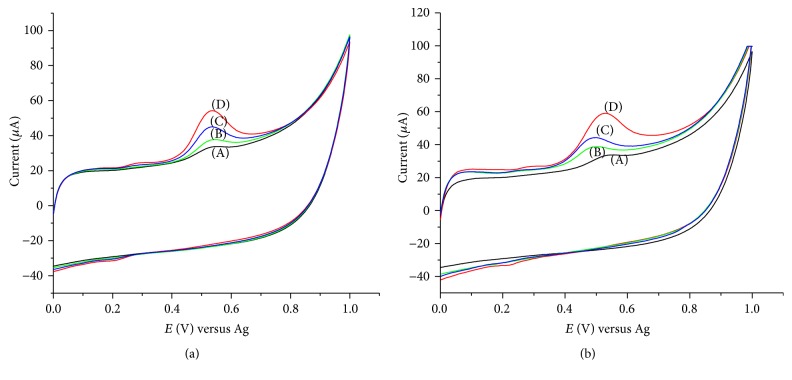
Effect of concentrations of 4-chlorophenol (a) and 2,4-dichlorophenol (b) on the response of 2 × 10^−5 ^M phenol at pH 6; phenol (A), 1 : 1 (B), 1 : 2 (C), and 1 : 5 (D) mixtures; scan rate: 0.05 V s^−1^.

Some phenolic compounds, such as 4-chlorophenol (CP) and 2,4-dichlorophenol (DCP), that coexist with phenol in environmental samples can interfere in the determination of phenols at SWCNTs/SWCNTs/PEDOT. In pH 6 buffer solution, the oxidation peak potential of phenol was 0.551 V and its peak current was 6.87 *μ*A. In the presence of one-to-one mixtures of CP and DCP, the peak potentials recorded were 0.546 and 0.502 V, respectively, while the peak currents were 11.13 and 11.28 *μ*A, respectively. The peak current was almost double compared to phenol alone, Figure 9. Further studies showed that the peak potentials of 2,4,6-trichlorophenol (TCP) and pentachlorophenol (PC) were 0.48 and 0.614, respectively, and the peak currents observed for one-to-one mixture were almost the same, but the current increases and the peak gets broad as the ratio increases. The presence of *p*-nitrophenol and *o*-nitrophenol has no effect on a one-to-one mixture, but the peak current decreases as concentration ratio increases. Thus, *p*-nitrophenol and *o*-nitrophenol interfere in the determination of phenol only when present in concentrations much higher than phenol. The decrease in current response also indicates that the electrode cannot be applied for the detection of nitrophenols. The peak potentials for catechol and hydroquinone were less than 0.1 V and they showed no effect on the response for phenol. These results suggest that voltammetric determination of phenol in environmental samples is free of interference from catechol and hydroquinone; however, it may suffer from that of 4-chlorophenol and 2,4-dichlorophenol with higher concentrations of the other phenolic compounds.

## 4. Conclusions

SWCNTs were successfully incorporated in PEDOT and were used to modify bare SPCEs. The electrochemical oxidation of phenol, 4-chlorophenol, 2,4-dichlorophenol, and 2,4,6-trichlorophenol was successfully investigated at PEDOT/SWCNTs modified SPCE. The electrode demonstrated enhanced electron transfer properties and rapid response to phenol, and chlorophenols with good sensitivity. The well-known electrocatalytic properties of SWCNTs together with the good conductivity of PEDOT enhanced the sensing ability of SPCE. The study of the oxidation of the phenolic compounds presents an important finding in the preparation of SWCNTs/PEDOT modified SPCE sensor for the determination of phenolic compounds. The modified electrode was electrochemically characterized, optimized, and utilized for the determination of phenols and chlorophenols. The composites of electropolymerized polymers with CNTs used as modifiers significantly increased the electroactive surface area of the prepared electrode as indicated by the CV characterization steps.

The sensors developed were simple and efficient and possess a good operational stability and reproducibility. The analytical performance of the sensors made the quantification of phenolic compounds in water samples possible. Although the sensors cannot differentiate between the various phenolics present in the water samples analyzed, due to the recognition of similar functional group, they have the potential for assessing the phenolic statuses of water or other phenolic compound containing samples. However, further work is required in terms of improving the selectivity of the sensor, introduction of prior separations techniques, and the analytical applications of these sensors for the analysis of polyphenols in different samples.

## Figures and Tables

**Table 1:** Comparative potential and current responses for 1 × 10^−4^ M phenol at SPCE and modified SPCE.

Sensor	Peak potential (V)	Peak current (*μ*A)
SPCE	0.639	4.259
SPCE/SWCNTs	0.518	4.801
SPCE/PEDOT	0.602	8.228
SPCE/PEDOT/SWCNTs	0.580	7.182
SPCE/SWCNTs/PEDOT	0.509	21.38

**Table 2:** Dependence of the peak current on the scan rate.

Scan rate υ (Vs^−1^)	$\sqrt{\upsilon }$	Phenol	4-Chlorophenol	2,4-Dichlorophenol	2,4,6-Trichlorophenol
		*i* (*μ*A)	*i* (*μ*A)	*i* (*μ*A)	*i* (*μ*A)
0.02	0.141	8.23	20.79	14.92	8.49
0.05	0.224	13.66	32.59	26.97	18.77
0.08	0.283	17.66	39.74	38.15	28.48
0.10	0.316	19.13	41.05	43.51	33.41
0.13	0.361	22.40	46.19	49.90	38.54
0.15	0.387	23.05	51.06	54.72	42.98
0.18	0.424	24.21	55.70	57.65	45.99
0.20	0.447	26.23	58.73	59.35	44.99
0.25	0.500	28.43	65.07		
0.30	0.548	30.91	69.05		

**Table 3:** Response characteristics of the calibration graphs for the different phenolic compounds at SPCE/SWCNTs/PEDOT electrochemical sensor.

Phenolic compound	*i* (*μ*A) = *ac* (*μ*M) + *b*		Linear range (*μ*M)	Detection limit (*μ*M)	*R* ^2^
	Slope (*a*)	Intercept (*b*)			
Phenol	0.196	0.389	0.6–100	0.38	0.999
	0.046	1.699	200–600		0.996

4-Chlorophenol	0.339	1.283	0.8–100	0.28	0.996
	0.054	36.920	200–500		0.998

2,4-Dichlorophenol	0.341	1.681	0.5–60	0.16	0.998
	0.156	11.804	50–300		0.998

2,4,6-Trichlorophenol	0.311	1.668	0.4–100	0.16	0.998
	0.148	5.378	220–500		0.990

**Table 4:** Comparison of different electrodes for electrochemical detections of phenol and chlorophenols.

Phenolic substrate	Detector	Linear range (*μ*M)	Sensitivity (*μ*A/*μ*M)	Detection limit (*μ*M)	Reference
Phenol	*α*-Cyclodextrin (CD) modified screen printed graphite-based ink	5–100	2.11	4.6	[3]
	SPE/enzyme/immobilization ATChCl/H_2_O_2_/phenol	5–200	na	3.7	[31]
	Tyrosinase modified screen printed four-channel graphite-coated Au-array	2–40	0.93		[32]
	HRP-modified screen printed four-channel graphite-coated Au-array	2–300	0.50		[32]
	SPE/MWCNT/Bi/Tissue	2–200		1.17	[33]
	**SPCE/SWCNTs/PEDOT**	**0.6–600**	**0.196**	**0.38**	**This work**

4-Chlorophenol	Tyrosinase modified screen printed four-channel graphite-coated Au-array	5–30	1.610		[32]
	HRP-modified screen printed four-channel graphite-coated Au-array	5–60	3.380		[32]
	**SPCE/SWCNTs/PEDOT**	**0.8–500**	**0.339**	**0.28**	**This work**

2,4-Dichlorophenol	**SPCE/SWCNTs/PEDOT**	**0.5–300**	**0.341**	**0.16**	**This work**

2,4,6-Trichlorophenol	HRP-modified screen printed four-channel graphite-coated Au-array	2–140	0.21		[32]
	**SPCE/SWCNTs/PEDOT**	**0.5–300**	**0.311**	**0.16**	**This work**

**Table 5:** Recovery study.

	Sample containing phenol (*μ*M)	Added phenol (*μ*M)	Found phenol (*μ*M)	Recoveries (%)
Distilled water	10	30	40.75	101.88
	10	60	70.04	100.06
	10	70	80.85	101.06

Tap water	10	30	39.54	98.85
	10	50	61.43	102.38
	10	70	83.57	104.46
